# Diagnosis and treatment of SAPHO syndrome: A case report

**DOI:** 10.3892/etm.2014.1758

**Published:** 2014-06-04

**Authors:** XINGHUA SONG, WENWEN SUN, ZHAOWEI MENG, LU GONG, JIAN TAN, QIANG JIA, CHUNSHUI YU, TIELIAN YU

**Affiliations:** 1 Department of Nuclear Medicine, Tianjin Medical University General Hospital, Tianjin 300052, P.R.China; 2 Department of Rheumatology and Immunological Diseases, Tianjin Medical University General Hospital, Tianjin 300052, P.R.China; 3 Department of Radiology, Tianjin Medical University General Hospital, Tianjin 300052, P.R.China

**Keywords:** synovitis, acne, pustulosis, hyperostosis and osteitis syndrome, skin lesions, multiple imaging modalities

## Abstract

The present study reports a rare case of synovitis, acne, pustulosis, hyperostosis and osteitis (SAPHO) syndrome in an adult male. The 42-year-old man complained of skin lesions, chest pain and lumbago. Laboratory evaluations demonstrated an elevated erythrocyte sedimentation rate and increased levels of C-reactive protein. Computerized tomography, bone scintigraphy and magnetic resonance imaging revealed multiple bone lesions. A diagnosis of SAPHO syndrome was made. Non-steroidal anti-inflammatory drugs, alendronate sodium and steroids were administered, which resulted in clinical improvement. The current case study demonstrates that skin manifestation and multiple imaging modalities are important in generating a definite diagnosis of SAPHO syndrome, and that early treatment is vital for a positive outcome.

## Introduction

Synovitis, acne, pustulosis, hyperostosis and osteitis (SAPHO), is a rare syndrome that is mainly reported in the West and Japan (1), with few cases reported in China. Genetic susceptibility and infection with *Propionibacterium acnes* are the main pathogenic hypotheses for the syndrome. Apart from these, proinflammatory cytokines, including tumor necrosis factor α, are also suspected to be involved in SAPHO syndrome (1). SAPHO syndrome is relatively benign and symptomatic treatment is currently an effective management strategy. Early recognition and treatment is likely to improve the health and quality of life of patients with SAPHO. In the present case study, the diagnosis and management of a Chinese patient with SAPHO syndrome is described.

## Case report

**Figure 1:**
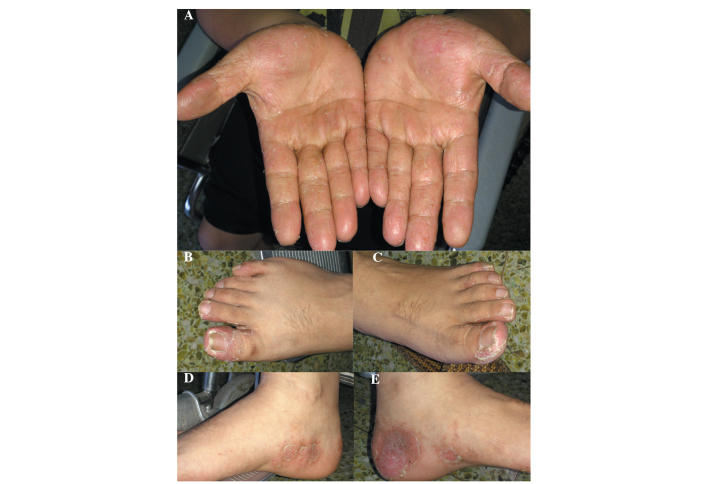
Physical examination on admission revealed bilateral psoriasis on (A) the palms and (B and C) the interdigital surfaces of the feet. (D and E) There were pustules on the inner surfaces of the ankles.

A 42-year-old Chinese male presented with bilateral psoriasis on the palms for two months without any clear predisposing cause. The patient later developed skin problems bilaterally on the ankles and interdigital surfaces of the feet. Following consultation with doctors in a local hospital, the patient was prescribed treatment for erythra; however, this did not lead to much improvement. The patient complained of chest pain and lumbago for three weeks, and experienced episodes of limited mobility in the mornings for ~15 min each time. Physical examination on admission revealed bilateral psoriasis on the palms and interdigital surfaces of the feet, and pustules on the inner surfaces of the ankles (Fig. 1). There was also redness, swelling and tenderness in the left sternoclavicular joint area and tenderness in the lower back. The straight-leg raise test for the left leg was positive.

Blood tests revealed an elevation of the erythrocyte sedimentation rate (49 mm/h, normal range 0–15 mm/h), levels of C-reactive protein (4.04 mg/dl, normal range <0.80 mg/dl) and D-dimer (1,080 ng/ml, normal range 0–500 ng/ml). Routine blood examinations also demonstrated slight increases in the number of leukocytes (10.14×10^9^/l, normal range 4.00–10.00×10^9^/l), neutrophilic leukocytes (7.09×10^9^/l, normal range 2.00–7.00×10^9^/l) and platelets (394×10^9^/l, normal range 100–300×10^9^/l). The rheumatoid factor and human leukocyte antigen B27 tests were negative. The results for all other laboratory tests that were carried out were within normal range.

**Figure 2:**
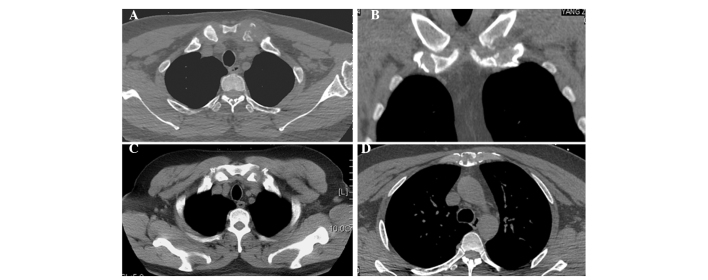
Computerized tomography (CT) scans of the sternum. The sternoclavicular joints revealed osseous erosions on (A–C) the left sternoclavicular joint area and (D) the manubrium.

**Figure 3:**
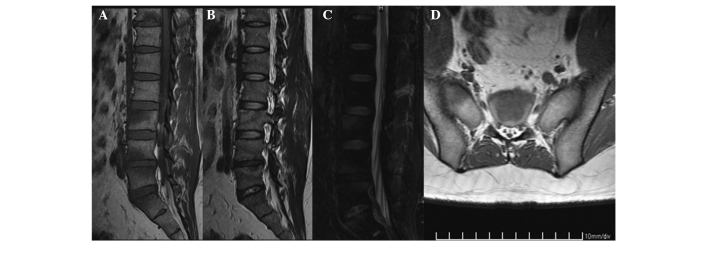
Sagittal magnetic resonance imaging (MRI) scans of the lumbar spine revealed diffused inflammatory bone changes in the T11, L3–L5 and S1 vertebral bodies that were (A) hypointense on the T1-weighted image, (B) hyperintense on the T2-weighted image and (C) hyperintense on the fat-saturated T2-fast spin-echo sequences. (D) Axial MRI of the sacroiliac joints demonstrated edema around the bilateral ala of the sacrum on the T1-weighted image.

**Figure 4:**
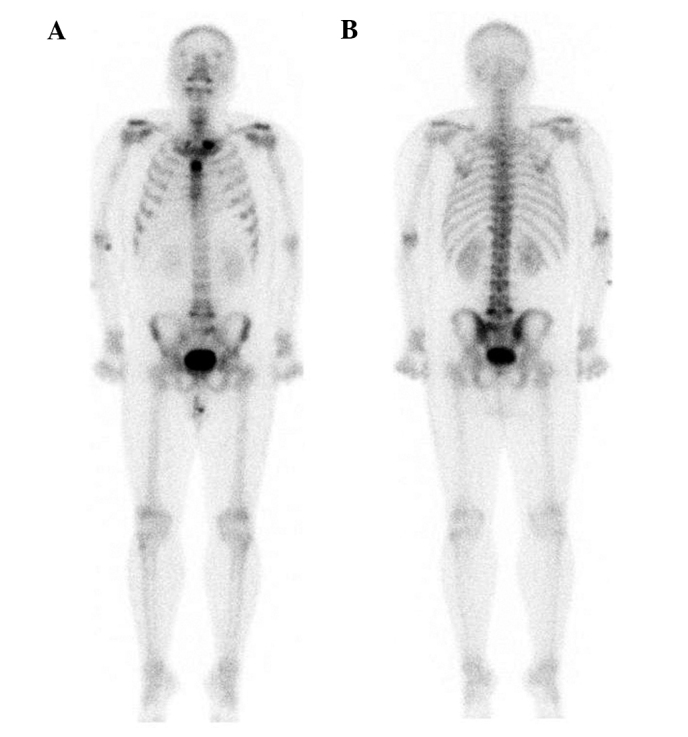
(A) Anterior and (B) posterior views of the whole body bone scan (WBS) revealed intense uptake at the proximal end of the left clavicle, manubrium sterni, fifth lumbar vertebra and right sacroiliac joint.

Computerized tomography (CT) scans of the sternum, sternoclavicular joints and sacroiliac joints revealed osseous erosions on the left sternoclavicular joint area, manubrium (Fig. 2) and bilateral sacroiliac joints. Magnetic resonance imaging (MRI) scans of the lumbar spine and sacroiliac joints demonstrated bone marrow edema at the levels of the T11, L3–L5 and S1 vertebra and the bilateral ala of sacrum (Fig. 3). To evaluate the skeleton, a whole body bone scan (WBS) was performed 3 h following the injection of 25 mCi ^99m^Tc-methylene-diphosphonate. Anterior and posterior views of the WBS revealed intense uptake at the proximal end of the left clavicle, manubrium sterni, fifth lumbar vertebra and right sacroiliac joint (Fig. 4).

A diagnosis of SAPHO syndrome was made according to the clinical manifestations (skin lesions and osteoarticular involvement), the results of the CT, MRI and WBS scans, and the analyses of the laboratory tests. Non-steroidal anti-inflammatory drugs (NSAIDs), alendronate sodium, leflunomide and steroids were administered, resulting in a notable remission of the clinical symptoms and the normalization of serum indices.

A written informed consent was obtained from the patient prior to publication.

## Discussion

The term SAPHO syndrome was first proposed by Chamot *et al* in 1987 (2), to describe a group of conditions that had similar osteoarticular involvement (osteitis mainly affecting the anterior chest wall) and that were frequently associated with different forms of dermatological manifestations. Studies have shown that from its onset, SAPHO syndrome is associated with an elevated erythrocyte sedimentation rate and increased C-reactive protein values (3–5). The etiopathogenetic mechanism of SAPHO syndrome remains unclear, although several hypotheses have been proposed involving bacteriologic, immunologic and genetic factors. By combining bacteriologic, immunologic and genetic data, Hayem (1) considers SAPHO syndrome as a ‘reactive osteitis’, namely a pathogenic sequence in which the opportunistic organism (*Propionibacterium acnes*) takes advantage of genetically determined deficiencies in antibacterial defense mechanisms and subsequently induces auto-amplification of the inflammatory response, possibly with an autoimmune component.

The diagnosis of SAPHO syndrome is based on history, characteristic scintigraphic and radiological results, and skin manifestations. Any one of the following criteria is regarded as sufficient to diagnose SAPHO syndrome: i) multifocal osteitis with or without skin lesions; ii) sterile acute, subacute or chronic arthritis associated with pustular psoriasis, palmoplantar pustulosis, acne or hidradenitis suppurativa; and iii) sterile osteitis combined with one of the skin manifestations (6). However, the dermatological and skeletal conditions do not always occur in parallel, and they may be separated by a number of years. Thus, diagnosing SAPHO syndrome is difficult in certain cases, particularly if the dermatological manifestations are absent (7).

WBSs using ^99m^Tc-methylene-diphosphonate are important for the diagnosis of SAPHO syndrome, particularly for detecting multiple and early bone involvement. Bone scintigraphy is a sensitive imaging modality that is able to identify uptake in characteristic regions when changes in radiography are absent or subtly abnormal (6). The sternoclavicular junction is the most common site of involvement in adults, followed by the spine and sacroiliac joints (7). In the present case study, all the common sites were involved to a certain extent. The radiological results of SAPHO syndrome consist of osteolysis, osteitis, hyperostosis and osteosclerosis (8) Osteolysis is occasionally observed, particularly in the initial stages of the disease (7,9), as is the case in the current study. CT scans provide a detailed depiction of the osteoarticular lesions and are the primary imaging modality of the chest wall, particularly for the sternoclavicular region. MRI scans are recommended in cases of spondylodiscitis in SAPHO syndrome in order to provide a better understanding of the extent of the inflammatory process. This is due to the fact that chronic sclerotic bone lesions exhibit low signal intensity in T1- and T2-weighted images, whereas active lesions appear hypointense on T1- and hyperintense on T2-weighted images (8). MRI results reveal SAPHO vertebral lesions, including body corner erosions, signal abnormalities in the contiguous vertebrae and the narrowing of disk spaces (10).

Until now, there have been no treatment guidelines for SAPHO syndrome. Current treatment of this illness is multimodal and empirical, and is mainly focused on relieving symptoms. NSAIDs, with or without antibiotics, are the primary treatment (11). Several studies have supported the effectiveness of bisphosphonates as a treatment for SAPHO syndrome as they exhibit a good cutaneous and articular response. These drugs not only take part in bone remodeling but also have anti-inflammatory properties that inhibit cytokine secretion by macrophages (12–14). In the present case study, NSAIDs, oral bisphosphonates, leflunomide and steroids were prescribed simultaneously. According to the results observed in the patient, this combination proved to be effective for the treatment of SAPHO syndrome, and terminated its rapid onset.

The current study described a rare case of SAPHO syndrome with skin problems, musculoskeletal involvement of the anterior thoracic wall, lumbar vertebra and sacroiliac joint erosion. The present case highlights the importance of using multiple imaging modalities to produce a definite diagnosis of SAPHO and indicates that early treatment of SAPHO is vital for a positive outcome.
